# Highly Ordered T6 Organic Semiconductor Networks on MoS_2_ Nanosheets for Optoelectronic Applications

**DOI:** 10.1021/acsanm.5c05073

**Published:** 2026-02-04

**Authors:** Nicolò Galizia, Pasquale Orgiani, Claudia Cardoso, Deborah Prezzi, Antonio Cassinese, Riccardo Frisenda

**Affiliations:** † Physics Department, Università Napoli Federico II, P.le Tecchio 80, Naples 80125, Italy; ‡ CNR-IOM Istituto Officina Dei Materiali, Basovizza I-34149, Trieste, Italy; § AREA Science Park, Località Padriciano I-34149, Trieste, Italy; ∥ CNR-NANO Istituto Nanoscienze, Centro S3, Modena I-41125, Italy; ⊥ Physics Department, Sapienza University of Rome, Piazzale Aldo Moro 5, Rome 00185, Italy

**Keywords:** van der waals epitaxy, sexithiophene, MoS2, 2D materials, DFT

## Abstract

Van der Waals 2D crystals, with their dangling bond-free surfaces and extremely low roughness levels, are highly appealing substrates for the epitaxial growth of organic semiconductors. The growth has important consequences in the fabrication of organic electronic components such as organic light diodes. Here, using a MoS_2_ nanosheet, an n-type semiconducting 2D material, as a substrate, we grow highly ordered crystalline needles made of sexithiophene (T6), a p-type organic semiconductor. Using atomic force microscopy topographic analysis, X-ray diffraction, and micro-Raman spectroscopy, we show that the T6 needles show both short- and long-range order thanks to an alignment between the T6 long axis and high symmetry directions in MoS_2_. By statistical analysis we demonstrate that the T6 needles show a small mismatch of ±7**°** between their long axis and the zigzag directions of MoS_2_. Interestingly, T6 grown on multilayer graphene does not show such an order, resulting in a randomly oriented needle network, while T6 grown on atomically thin MoS_2_ still show long-range order. Density functional theory predicts an alignment of the T6 long axis along the zigzag direction of MoS_2_ to minimize the total energy of the system by maximizing the number of thiophene rings positioned on top of sulfur atoms from MoS_2_. The ideal interface between T6 molecules and MoS_2_ also has implications in the charge transfer of photoexcited carriers as demonstrated by microphotoluminescence spectroscopy. These results demonstrate that van der Waals materials are ideal substrates for the growth of organic molecules and that subtle variations in the van der Waals long-range potential can influence the long-range order of the molecular crystals. The ordered one-dimensional T6 crystallites are interesting for polarized or anisotropic optoelectronic applications.

## Graphical abstract

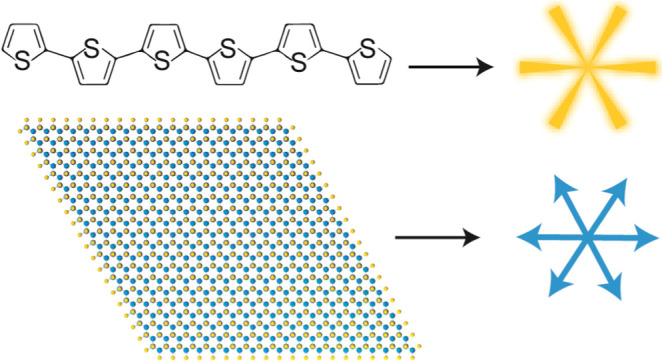

## 1 Introduction

The pursuit of mechanically flexible, lightweight, low-cost (opto-)­electronics has led researchers toward the development of organic semiconducting components, which led to commercial devices such as organic light-emitting diodes (OLEDs).^1–4^ Despite the large commercial success of OLEDs, for many large-scale applications, a severe optimization of device performance is still necessary.^5,6^ A key factor for high performance is the quality of the organic semiconductor thin films. In this respect, both the crystal quality and molecular orientation with respect to the substrate are crucial. In order to control the organic thin film structure, morphology, and quality, a plethora of organic semiconductor-substrate systems have been investigated. An especially appealing class of substrates for the growth of organic semiconductors are van der Waals (vdW) two-dimensional (2D) materials.^7–14^ In the literature, most of the examples of organic molecules on vdW materials focus on the growth of organic semiconductors on graphene^15^ or on boron-nitride (hBN),^16^ respectively, a (semi)­metallic and an insulating vdW material. In particular, successful ordered growth has been demonstrated in the case of parahexaphenyl, pentacene, C60, and dihydrotetraazaheptacene, among others.^17–22^ We also refer the reader to the review from Kratzer et al., where the adsorption and epitaxial growth of small organic semiconductors on hBN is discussed in detail.^23^ A special class of mixed-dimensional 0D-2D van der Waals heterostructures, which have not been investigated in detail yet, are organic semiconductors grown on vdW semiconductors.^24–27^ In this case, the resulting electronic structure is interesting for applications and manipulation of light and charge at the nanoscale and for the engineering of excitons’ potential landscape.^28–30^ The integration of 2D semiconducting materials with organic molecules presents significant potential for downsizing, offering excellent optoelectronic properties thanks to the atomically flat interfaces.^31^

Here, we present a new mixed-dimensional 0D-2D vdW heterostructure based on the growth of rod-like sexithiophene (T6) molecules on mechanically exfoliated single-crystal MoS_2_ nanosheets. In this mixed-dimensional 0D-2D structure, a p-type organic material (T6) is interfaced with an atomically thin n-type inorganic vdW semiconductor. By a combination of topographic measurements and microspectroscopies, we show that T6 on MoS_2_ forms a very ordered network of needles aligned along the zigzag directions of the MoS_2_ lattice. Such a needle growth was observed previously by Simbrunner et al. in the case of T6 on mica substrates, a family of vdW insulators, although with a more azimuthally disordered structure.^32^ This is in contrast to the disordered growth of T6 on SiO_2_, which was compared directly in the same sample thanks to the micrometric nature of MoS_2_ flakes. Density functional theory (DFT) calculations, performed on T1 and T6 molecules on MoS_2_, suggest that T6 molecules tend to align along MoS_2_ zigzag directions to maximize the number of thiophene rings sitting on top of S atoms from the underlying MoS_2_ lattice and thus minimize the total energy. Experimentally, the short- and long-range order of T6 on MoS_2_ also allows for precise Raman spectroscopic characterization of T6 vibrational modes even at room temperature, which is not the case for T6-SiO_2_. Finally, microphotoluminescence (PL) spectra reveal an interaction between the excited electronic states of T6 and the bands of MoS_2_, where charge transfer phenomena of the photogenerated electrons and holes reveal the intimate contact between the two materials.

Our findings offer valuable insights into the interface between MoS_2_ and T6, which could serve as a foundation for understanding how rod-like conjugated molecules grow on vdW semiconductors. By controlling the size and ordering of crystallites in self-assembled networks, future electronic and optoelectronic devices can be designed to exploit the intrinsic properties of organic semiconductors at the van der Waals interface. Applications requiring highly ordered one-dimensional crystallites could enable features such as polarized light emission, anisotropic optical properties, or vertical charge transport within organic semiconductors.

## 2 Results and Discussion

The growth and morphology of T6 organic molecules are known to depend on the substrate on which it is performed. In fact, the interface between molecules and substrates is one of the driving forces in the self-assembly of crystallite networks. In the case of vdW materials, where the molecules grow by vdW epitaxy,^7^ the symmetry of the substrate plays a crucial role, as reported in previous works.^19^ For the analysis of the growth of T6 on mechanically exfoliated crystalline MoS_2_ flakes, we use various complementary morphological and spectroscopic techniques. A scheme of T6, MoS_2_, and the evaporation system based on a Knudsen cell containing T6 material are shown in Figure 1a. Figure 1b shows microscope photographs of a mechanically exfoliated MoS_2_ multilayer flake (thickness ≈ 200 nm) transferred onto a SiO_2_/Si substrate. The photographs show, respectively, the flake directly after the transfer (left panel), after the deposition of T6 molecules (10 nm film, center panel), and after soaking the sample in a hot acetone bath (right panel). A visual inspection shows that the exfoliated MoS_2_ flake presents long straight edges with well-defined angles (approximately 60° and 120°) between straight edges. It is well-known that MoS_2_ is highly likely to cleave along the zigzag direction, resulting in the angles between two straight edges being 60° or integer multiples thereof.^33,34^ After the T6 evaporation, a change in the optical appearance of the MoS_2_ flake and of the SiO_2_/Si substrate is evident. Looking at the flake, a collection of needle-like black features can be identified already from the optical image. Finally, after the acetone bath, the needle-like features disappear and the flake appearance goes back to the pristine case, suggesting that the deposited T6 molecules have been removed from the sample.

**Figure 1:**
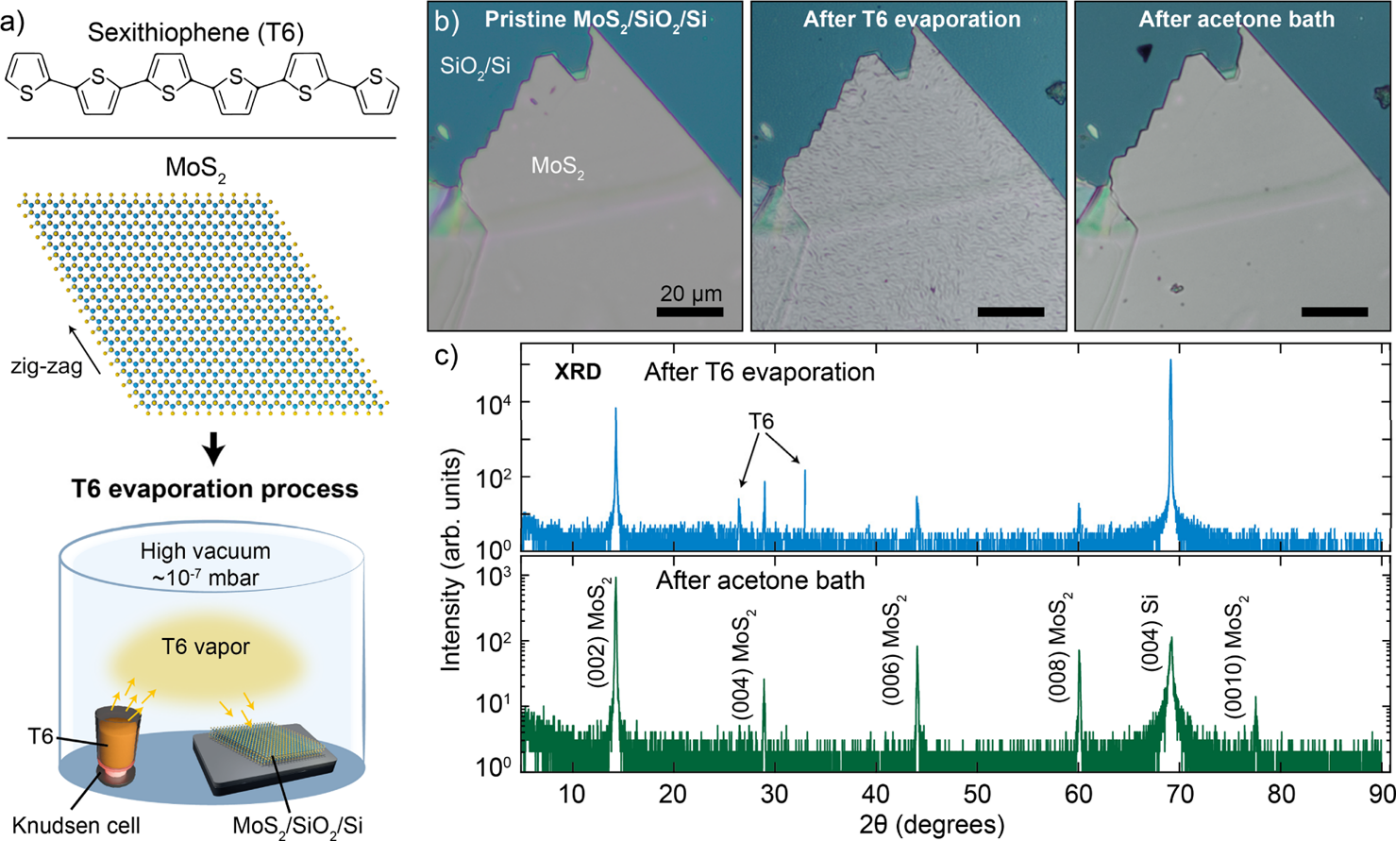
Highly ordered networks of T6 growth on MoS_2_mechanically exfoliated flakes. (a) Scheme of a T6 molecule, a top view of MoS_2_ crystal lattice, and the T6 evaporation process. (b) Optical microscope photographs of a mechanically exfoliated MoS_2_ flake transferred onto a SiO_2_/Si substrate (left), after the deposition of T6 molecules (center), and after dipping the sample in an acetone bath for 15 min (right). (c) X-ray diffraction from the MoS_2_ flake with T6 grown on top (black line) and from the same flake after the acetone bath.

To probe the crystalline order of the MoS_2_ flake and of the T6 deposited on top of it, we perform an X-ray diffraction experiment on the T6/MoS_2_ sample before and after the acetone bath. Symmetrical θ–2θ scan of T6 epilayers grown on top of MoS_2_ flakes (Figure 1b) are reported in Figure 1c. Regarding the MoS_2_, the XRD spectrum (green curve) only contains (00l) peaks, thus indicating the preferential *c*-axis orientation of the flakes along their [001] crystallographic direction without any trace of other orientations and spurious phases (e.g., 1T-MoS_2_). By the position of the symmetric (002) Bragg reflections, the out-of-plane *c*-axis parameters were calculated to be (1.233 ± 0.001) nm, which corresponds, within the experimental error, to the bulk-like value of 1.2324 nm reported for the 2H–MoS_2_ single crystals.^35^ On the contrary, θ–2θ scan of the T6 epi-layer (blue curve) indicates a crystallographic orientation with triclinic phase characteristics (see also Figure S1 in the Supporting Information for a zoom in the T6 region).^36^

After having established the crystalline and long-range order nature of the MoS_2_ sample and of the T6 grown on top, we focus on the short-range order of the T6 crystallites. We use AFM topographic mapping to compare the growth of the T6 molecules onto the multilayer MoS_2_ flake and on the SiO_2_/Si. Figure 2a shows a 50 × 50 μm^2^ AFM topography map of the sample. This image shows the azimuthally ordered T6 network on top of the MoS_2_. Yellow contour lines highlight the straight edges of the MoS_2_ flake, which are zigzag directions of the crystal lattice. The T6 needles appear aligned along three particular directions roughly parallel to the MoS_2_ zigzag directions; a more accurate analysis of these angles will be presented later in the paper. From the AFM image, it is also clear the striking difference between the ordered network of T6-MoS_2_ and the less ordered growth of T6-SiO_2_, visible as islands with not well-defined shapes.

**Figure 2:**
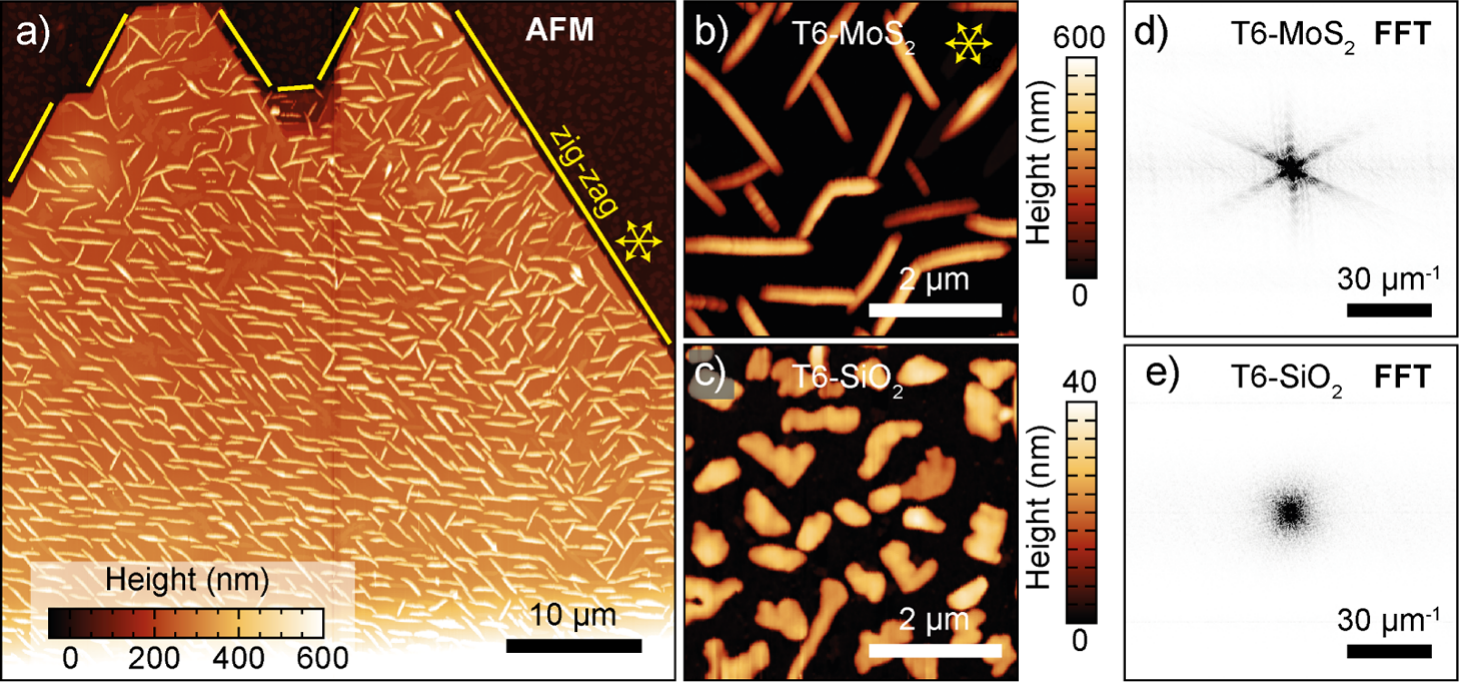
Topographic analysis of T6 growth on MoS_2_. (a) AFM topography recorded in a 50 × 50 μm^2^ region of the MoS_2_ flake shown in Figure 1. The MoS_2_ flake zigzag directions are indicated by yellow lines. (b,c) High-resolution AFM topography of the T6 structures formed on the MoS_2_ flake (b) and on the SiO_2_ part (c). (d,e) FFT of the topographic images in panels (b) and (c).

The ordered/disordered growth of T6 on MoS_2_ and on SiO_2_ is even more evident when focusing on AFM mappings of smaller regions of 5 × 5 μm^2^, as shown in Figure 2b (T6-MoS_2_) and 2c (T6-SiO_2_). In the case of T6-MoS_2_ needle-like structures are visible aligned along three well-defined directions. For T6-SiO_2_, the more random nature of the structures formed is evident. Interestingly, the heights of the T6 structures in the two substrates are also different, with the T6-MoS_2_ structures being significantly taller than the T6-SiO_2_ structures, as can be seen by looking at the color scale of Figure 2b,c. By performing a two-dimensional fast Fourier transform (FFT) of the topographic mapping just discussed, we can assess again the presence of azimuthal order in the T6-MoS_2_ case, absent in the T6-SiO_2_ case. Figure 2d,e shows the Fourier transform of the maps from Figure 2b,c computed by the fast Fourier transform algorithm. The FFT in the T6-MoS_2_ present six rays with angles of 60° between them, radiating from the center of the image and indicating long-range order in the T6 network (see also Figure S2 in the Supporting Information for the FFT of Figure 2a). Such a long-range order is not to be intended as a perfect translational order typical of single crystals but rather as a less strict one where, thanks to their relative alignment, different molecules belonging to different needles can have a similar orientation even at tens of micrometers of distance. In the case of T6-SiO_2_, the FFT only shows a central spot, indicating that the AFM image does not contain spatially ordered features.

In order to get more information about the order of the T6 needles network, we analyze the angles of all the needles from Figure 2a. In the needle’s angular distribution analysis, the direction of each needle was considered with respect to the *x*-axis of the figure and then measured counterclockwise. The *x*-axis of the figure is also roughly aligned with one of the zigzag directions of the MoS_2_ flake. Figure 3a shows a histogram built from the angles of 1275 T6 needles. In the histogram, we also highlighted the positions of the zigzag and armchair angles of the flake. As can be seen, most of the investigated needles align well with the zigzag directions and not with the armchair ones. This indicates a favorable alignment of the molecular needles along zigzag high-symmetry directions in MoS_2_, highlighting the important role of the substrate symmetry in the van der Waals epitaxy growth. A closer look at the histogram also reveals that the orientation of the needles is not exactly along the zigzag directions, but there is a misalignment of approximately ±7°. Such a feature resembles the misalignment of ±5° observed by Matkovic and coauthors^19^ for parahexaphenyl (6P) needles grown on hBN, which the authors attribute to a collective, epitaxial effect driven by molecule–molecule interactions once bulk-like 6P crystallites form, rather than by an isolated molecule–substrate alignment. This explanation applies also to our case, with one key difference being that at the DFT level, a 6P molecule tends to align along the armchair direction of hBN, while in our case, a T6 molecule aligns along the zigzag direction of MoS_2_. Apart from the angle of the needles, we also extracted their length, and Figure 3b shows a histogram of the length of the 1275 needles. The histogram shows an homogeneous length distribution, with most of the needles being between 1 and 2 μm long. Finally, in Figure 3c, the height of all the analyzed needles is scatter plotted as a function of their length, showing that the height is approximately independent of the needle length.

**Figure 3:**
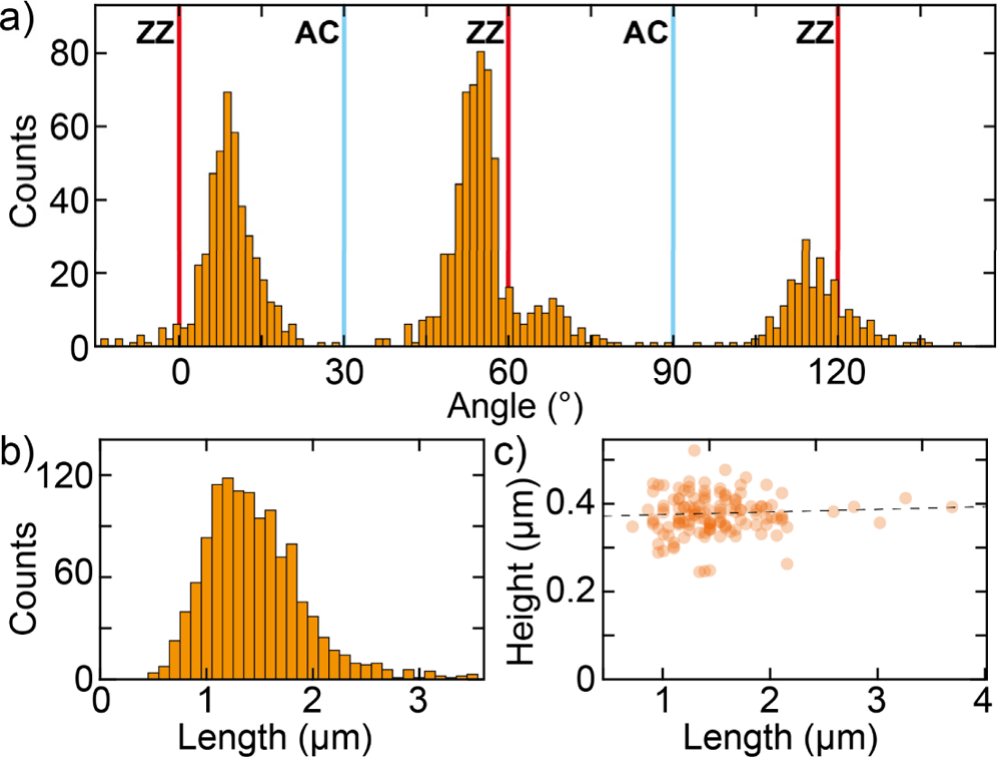
Angle and size distribution of T6 needles. (a,b) T6 needle angle (a) and length (b) histograms extracted from the AFM image in Figure 1b analyzing 1275 T6 needles. In the angle histogram, the red and light blue vertical lines indicate the zigzag and armchair directions of the MoS_2_ flake. (c) Scatter plot of the needles’s heights and lengths. The dashed line is a linear fit to the data.

To better understand the origin of the observed selective alignment on MoS_2_, we computed the stability of both the single thiophene molecule (T1) and T6 on MoS_2_ by performing several DFT calculations at varying the registry (see the methods for details). Figure 4a shows the relative stability of the different configurations of T1 with respect to the minimal energy (set to zero), where T1 is placed on top of an S atom and aligned along the armchair direction. The energy of this configuration differs by only a few meV from other S-atop configurations (orange circles in Figure 4a), which were built to have a zigzag alignment and the S atom either at the center of the MoS_2_ hexagon or on top of a Mo atom. Configurations with the center of T1 being at the center of a MoS_2_ hexagon (hollow configurations, blue circles) or on top of a Mo atom (Mo-atop, lilac circles) are overall less stable than the S-atop configurations, lying ∼70–120 meV higher in energy depending on the specific orientation of the molecule. Interestingly, the least stable structures are those featuring an S atom of T1 on top of a S atom of the substrate, while the most stable are the S-atop. The results for the single molecule can be used as a rough guide to explore significant configurations for T6, considering the energy additive at the first approximation. Figure 4b shows a configuration where T6 is aligned along the zigzag direction of MoS_2_ and we maximized the number of S-atop registries for the thiophene units (two of them, highlighted by orange areas). If we do the same for the armchair alignment (panel c), we can accommodate only a single S-atop unit but also end up with one of the least stable configurations (highlighted by a blue circle in panel c). In agreement with our rule of thumb derived from single molecule calculations, the T6 configuration with zigzag alignment is about 115 meV more stable than the one with armchair alignment, in agreement with our rule of thumb derived from single molecule calculations, i.e., comparable to the energy difference between the most and the least stable configurations for T1. To further test this picture, we have also considered a second zigzag configuration that maximizes the number of least stable registries, i.e., two thiophene units where the S is on top of an S atom of MoS_2_. In this case, T6 slides on the surface upon structural optimization, reverting to the previous configuration with two S-atop units. In addition to explaining the selective growth for T6, these calculations also suggest a length dependence for thiophene-based oligomer T_*n*_ on MoS_2,_ with an onset at *n* = 5 because only from the fifth ring onward can the zigzag alignment accommodate two S-atop registries (Figure 4b). Consistently, a T4 oligomer (which lacks this second S-atop registry) oriented along the zigzag direction is only 19 meV more stable than one oriented along the armchair direction.

**Figure 4:**
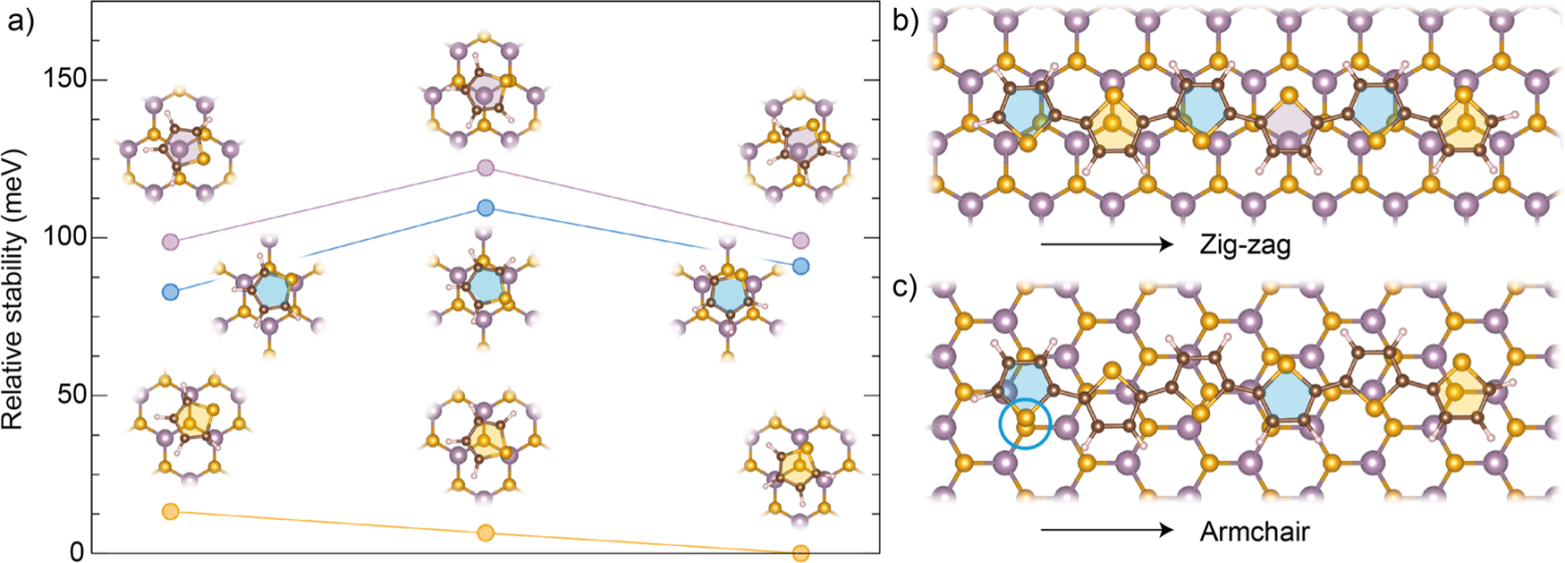
Stability of T1 and T6 on MoS_2_from DFT. (a) Relative stability of the thiophene molecule (T1) on MoS_2_. Orange data points indicate S-atop configurations, lilac points represent Mo-atop configurations, and blue ones stand for the hollow registry. Data points are accompanied by ball-and-stick models of the different configurations, where S atoms are represented by orange balls, C is brown, H white, and Mo lilac. (b,c) configurations with T6 being aligned along the zigzag (b) and armchair (c) direction of MoS_2_.

After having established multilayer MoS_2_ as a suitable substrate for the ordered growth of T6 needle-like structures, we focus on the investigation of the influence of the vdW material type and of the 2D material thickness on the morphology of the T6 crystallites. Figure 5 shows T6 grown on Gr and MoS_2_ flakes, both transferred on the same substrate. Sample 1 and sample 2 refer to different substrates and T6 growths. Interestingly, despite the T6 molecules growing in both vdW substrates in a needle-like geometry, the needle network in the MoS_2_ case is well aligned with high-symmetry zigzag directions, while in the case of Gr, the needles do not show a particular orientation along special directions, and the final network seems more azimuthally disordered regarding the alignment. Thus, although both vdW substrates promote needle-like growth of T6, the markedly different azimuthal order indicates that, beyond the common 3-fold (C3) in-plane symmetry, the detailed interfacial potential, including registry-dependent adsorption and surface chemistry, plays a key role in determining the final needle orientation. On the other hand, the thickness of the MoS_2_ flake seems not to play an important role in the orientation of the network, as shown in Figure 6. Here, two thin MoS_2_ flakes (thickness <5 layers) are used as a substrate for the growth of T6 with a nominal film thickness of 3 nm. From the AFM mapping, it is clear that in both cases the T6 needles align well with the MoS_2_ high-symmetry directions, and only a few angles between the needles, which are all multiples of 60°, can be observed.

**Figure 5:**
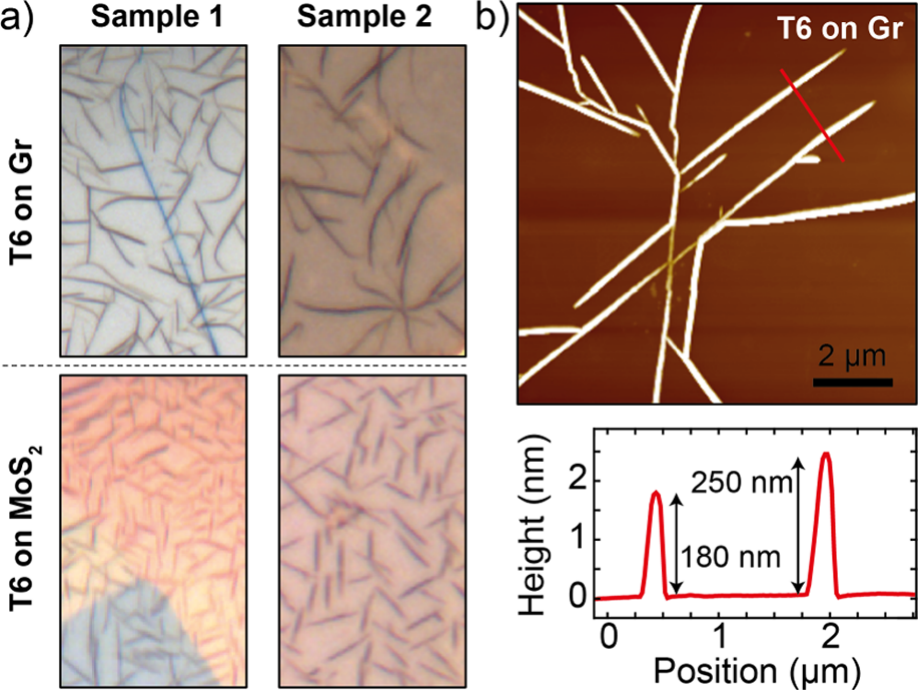
Comparison of T6 growth on Gr and MoS_2_flakes. (a) Optical microscope photographs of T6 grown on different samples of mechanically exfoliated graphite (top) and MoS_2_ (bottom) flake transferred onto a SiO_2_/Si substrate. (b) AFM topography of T6 needles grown on Gr.

**Figure 6:**
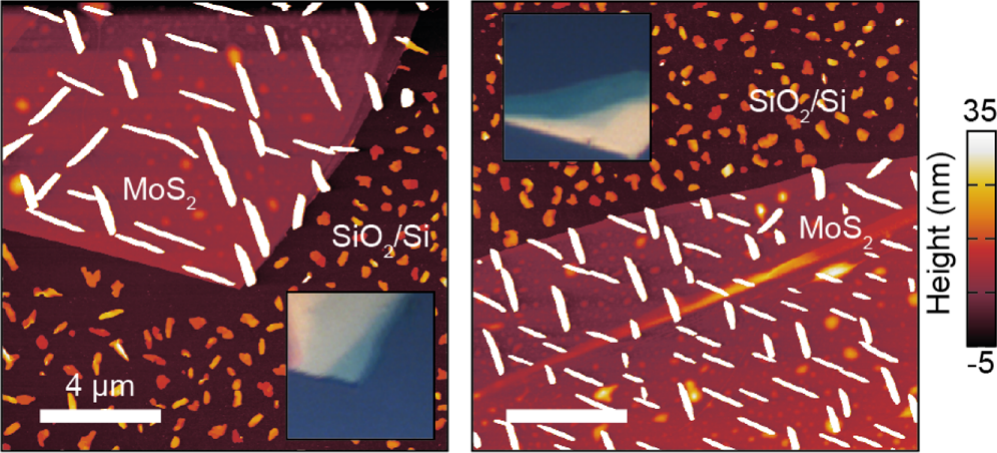
T6 growth on ultrathin MoS_2_flakes. AFM topography of T6 grown on two thin MoS_2_ flakes. Insets: Optical microscope photographs of the flakes.

Ultrathin MoS_2_ is known to be an intense bright optical emitter thanks to the direct bandgap in the monolayer and an indirect-to-direct transition when reducing its thickness to a few layers. We thus focus on the investigation of the optical properties of the few-layer MoS_2_ T6 sample, with a focus on the Raman vibrational properties and on the photoluminescence signal. Figure 7 shows two μ-Raman spectra acquired at 532 nm excitation on top of a T6 crystallite on MoS_2_ and on SiO_2_. Note that the two spectra have not been normalized or vertically offset. Comparing the two spectra reveals clear differences in both the peaks present in each spectrum and the shape of the low-frequency background. The T6-MoS_2_ Raman spectrum (blue curve in Figure 7) shows peaks at 384.9 cm^–1^ and 408.1 cm^–1^ assigned to the E_2g_ and A_1g_ vibrations in 3L MoS_2_. The frequency difference between these two peaks of 23.2 cm^–1^ allows one to univocally assign a thickness of three layers to the MoS_2_ flake. The peak at 521 cm^–1^ comes from the Si substrate. Finally, a tiny peak at 1049.7 cm^–1^ and two asymmetric peaks at 1460.9 cm^–1^ and 1511.5 cm^–1^ are consistent with vibrational frequencies reported previously in T6 single crystals.^37–39^ The strong Raman signal of T6 grown on MoS_2_ suggests that the molecules’ growth on this substrate is crystalline in nature with a microscopic order in addition to the mesoscopic long-range order of the T6 needle network discussed previously in the article. Interestingly, the Raman spectrum acquired on the T6-SiO_2_ crystallites (red curve in Figure 7) shows the absence of vibrational modes coming from the T6 molecules, and only peaks assigned to the Si substrate can be observed. Apart from the presence of individual peaks, the background in the T6-MoS_2_ and T6-SiO_2_ cases is very different. In the latter case, a broad background increasing at larger Raman shifts can be clearly observed, which is absent in the former case, where the background is flat and low in intensity. The background in the T6-SiO_2_ sample is due to photoluminescence from the T6 molecules, and it will be discussed in detail in the next paragraph. Nevertheless, the presence of such a signal from T6 indicates that the experiment is probing T6 molecules and not a bare SiO_2_/Si substrate, where such a background would be absent, thus proving that the Raman signal from T6 is strongly dependent on the substrate and thus on the morphology of the growth of T6.

**Figure 7:**
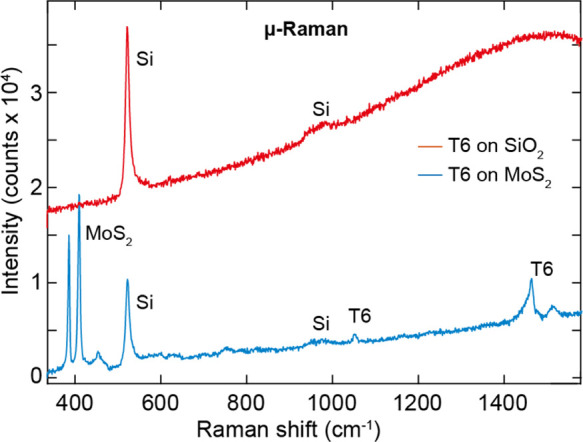
Micro-Raman spectroscopy of T6 on MoS_2_and SiO_2_. Micro-Raman spectra acquired with a 532 nm laser of T6 grown onto an MoS_2_ flake deposited onto a SiO_2_/Si substrate (blue line) and onto SiO_2_/Si (red line), where the individual peaks have been attributed to the different species. The two spectra are neither normalized nor offset.

After comparing the vibrational signal from T6 grown on 3L MoS_2_ and on SiO_2_/Si, we move to the discussion of the photoluminescence. Figure 8a shows a series of μ-PL spectra acquired at different positions on the sample (see inset) moving the laser spot in steps of 1 μm going from the 3L MoS_2_ to the SiO_2_/Si. In the initial spectra, the PL shows broad photoluminescence peaks between 1.8 and 2.2 eV coming from T6 and MoS_2_ and, at higher energies (close to the laser excitation energy), the Raman peaks discussed previously. A clear evolution of the PL signal can be observed when moving from T6-MoS_2_ to T6-SiO_2_, where the signal at 1.8 eV is reduced and a new peak appears around 2.15 eV. Also, the Raman peaks of T6 present an evolution similar to the discussion in the previous paragraph. Figure 8b compares the PL spectra of T6-MoS_2_ and T6-SiO_2_, where the dashed lines indicate the peaks that are observed in both spectra, and the PL spectra of pristine MoS_2_ and SiO_2_. The PL spectrum of T6-SiO_2_ shows four broad peaks located approximately at 1.78 eV, 1.96 eV, 2.02, and 2.12 eV, which are completely absent in the pristine SiO_2_ spectrum (orange curve and Figure S3 of the Supporting Information). Interestingly, the first, second, and fourth peaks are separated by an average energy of 0.17 eV, which is very close to the energy of the vibration at 1460.9 cm^–1^ (0.18 eV). These three peaks are in agreement with previous reports of T6 on SiO_2_ and in a single crystal, consistent with T6 molecule–molecule interaction.^40,41^ Their origin is most likely vibro-electronic, where electronic levels couple to vibrational levels. On the other hand, the peak at 2.02 eV was not observed in previous reports, and it could belong to another vibro-electronic series involving another phonon mode such as the one at 1050 cm^–1^ (0.13 eV).

**Figure 8:**
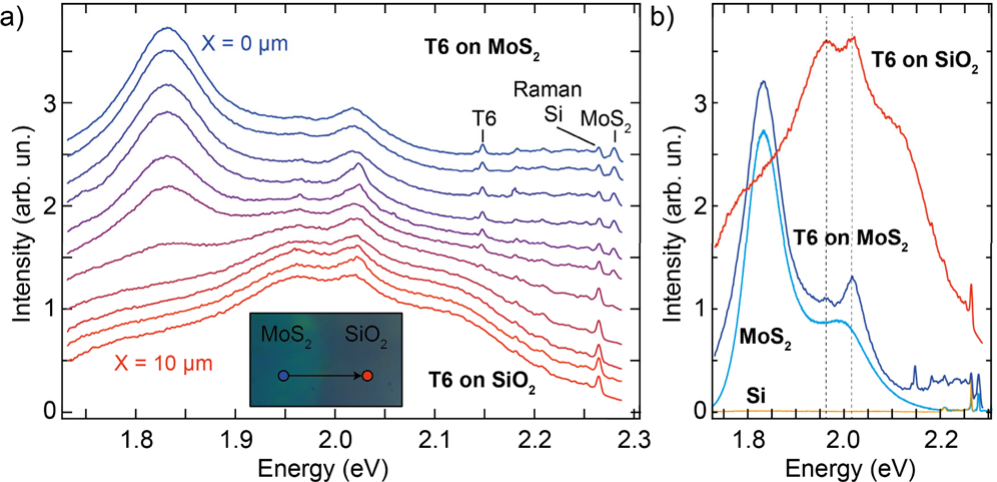
Photoluminescence emission from T6 on MoS_2_ and SiO_2_. (a) Microphotoluminescence spectra at 532 nm excitation as a function of distance going from T6 grown on a MoS_2_ flake (*X* = 0 μm) to T6 on SiO_2_ (*X* = 10 μm) recorded in steps of 1 μm. Inset: optical microphotograph of the sample with the path followed during the measurement shown. (b) Microphotoluminescence spectra at 532 nm excitation recorded on T6-MoS_2_ (dark blue) and T6-SiO_2_ (red). The dashed lines highlight the peaks present in both spectra. As a comparison, the spectra of pristine MoS_2_ (light blue) and pristine Si (orange) are also shown.

Usually the PL signal from molecules, and in particular from T6, strongly depends on the molecule–molecule and molecule–substrate interaction.^42^ In the case of the MoS_2_ substrate, the T6 PL is modified in respect to the SiO_2_ substrate. Here, we observe the absence of the peak at 2.12 eV and a global reduction of the PL emission intensity of the other peaks. Nevertheless, the peaks at 1.96 and 2.02 eV are still well resolved, while the peak at 1.78 eV is not visible. This can be due to the fact that in T6-MoS_2_ the PL spectrum most intense peak is the one centered at 1.83 eV, which can be attributed to the PL of 3L MoS_2_ (see the PL from the pristine flake reported as the light blue solid curve) and, specifically, to the direct bandgap radiative recombination of A excitons at the *K* point of the Brillouin zone in 3L MoS_2_. Overall, comparing the PL from pristine MoS_2_ and from T6-MoS_2_, one can see that the PL emission in T6-MoS_2_ is strongly dominated by the MoS_2_ emission, suggesting that the radiative recombination of electron–hole pairs is primarily happening in the MoS_2_ layer rather than in the T6 layer. Finally, we notice that the PL peak intensities do not strictly follow the expected decay rules. Such behavior can be attributed to the PL emission in the T6 needles being more strongly influenced by interfacial effects and molecule–molecule processes, rather than purely intrinsic molecular relaxation dynamics, as observed, for example, in close-packed T6 single crystals.^40^

The observed quenching of the T6 emission peaks in the T6-MoS_2_ system strongly suggests efficient charge transfer processes due to the energetics of the T6-MoS_2_ interface. In fact, MoS_2_ has a work function of approximately 4.6–4.9 eV and T6 exhibits HOMO and LUMO levels at −5.1 eV and −2.8 eV, respectively. Thus, a Type II band alignment is likely formed, where T6 would be an electron donor layer and MoS_2_ an acceptor.^43–45^ This configuration would facilitate electron transfer from photoexcited T6 to the MoS_2_ conduction band, while holes could be transferred from MoS_2_ to the T6 HOMO level, explaining the selective quenching of higher energy T6 states. A similar charge transfer process was recently reported by Xiong et al. for the organic/2D heterostructure composed of vanadyl phthalocyanine and WSe_2_, where a Type II band alignment was reported and exciton dynamics was shown to depend on the molecular configuration.^46^ Zhu et al. also made similar observations for tetracene on WS_2_.^47^ In our case, the efficiency of the charge transfer process could be higher for the T6 2.12 eV state than for the other states. In particular, MoS_2_ has a strong excitonic peak around that energy (B exciton), which matches the energy of the molecular state. Further experiments varying temperature and excitation power could reveal the activation energies for charge transfer processes and distinguish between different quenching mechanisms.

## 3 Conclusions

The study demonstrates that van der Waals semiconducting MoS_2_ nanosheets are excellent substrates for the epitaxial growth of highly ordered organic semiconductor networks such as sexithiophene needles. These T6 needles grown on MoS_2_ exhibit both short- and long-range order, aligning preferentially along the zigzag directions of the MoS_2_ lattice, as confirmed by atomic force microscopy, X-ray diffraction, and spectroscopic analyses. Density functional theory calculations reveal that this alignment minimizes the total energy of the system by maximizing the number of thiophene rings positioned on top of sulfur atoms in MoS_2_, providing a mechanistic understanding of the observed order. In contrast, T6 grown on multilayer graphene does not display such order, resulting in azimuthally randomly oriented networks, which highlights the critical influence of substrate symmetry and chemistry on organic crystal growth. The thickness of the MoS_2_ substrate, from multilayer to atomically thin, does not significantly affect the alignment of T6 needles, indicating the robustness of the ordering effect across different MoS_2_ thicknesses. Optical characterization, including Raman and photoluminescence spectroscopy, reveals strong interactions at the T6/MoS_2_ interface, with evidence of charge transfer phenomena and modified emission properties compared to T6 on SiO_2_. These findings provide valuable insights for the design of mixed-dimensional organic-2D semiconductor devices, where control over molecular ordering at the vdW interface can enable advanced optoelectronic functionalities.

## 4 Materials and Methods or Experimental

### 4.1 Sample Fabrication

Starting from a bulk MoS_2_ crystal (Moly Hill mine, Quebec, Canada), atomically thin flakes are deposited onto an elastomeric substrate (Gel-Film WF X4 6.0, GelPak). By a deterministic transfer method, we have deposited the MoS_2_ flakes onto a SiO_2_/Si substrate by pressing and gently releasing the Gel-Film against the substrate. T6 films were vacuum evaporated using a Knudsen cell and a base pressure of ≈1×10^–7^ mbar. The deposition rate and thickness were monitored by a quartz oscillator.

### 4.2 X-Ray Diffraction

The X-ray diffraction experiments were conducted using a Malvern Empyrean four-circle diffractometer with a Cu anode as the X-ray source, operating at 40 kV and 30 mA. A double-bounce 2xGe(220) hybrid monochromator, providing the Kα1 (1.540598 Å) wavelength only, combined with a GaliPIX3D detector with a 481 × 465 pixels active window (corresponding to equatorial and axial sizes of 6.8898° and 5.7632°, respectively), was employed.

### 4.3 Atomic Force Microscopy

Topographic measurements were performed in air with a Park Systems XE100 AFM (Suwon, Republic of Korea) in the tapping mode by using a Nanosensors PPP-NCHR AFM tip (Neuchatel, Switzerland) by collecting micrographs at a cantilever scanning rate of 0.1 Hz (speed between 0.5 and 5 μm/s).

### 4.4 Micro-Raman and Micro-PL Spectroscopies

The μ-Raman and μ-PL experiments were performed in a backscattering configuration by using a 532.2 nm laser excitation in a ultrahigh vacuum, as reported in detail in our previous publication.^48^ The spectrograph and monochromator (SpectraPro HRS-500, Princeton Instruments) were equipped with a 1200 grooves/mm grating during the μ-Raman acquisition and with 300 grooves/mm for the μ-PL.

### 4.5 DFT Calculations

Structural optimization of T1 and T6 on MoS_2_ was carried out by employing an ab initio implementation of DFT based on plane-waves and pseudopotentials, as available in the Quantum ESPRESSO (QE) package.^49,50^ Calculations were performed by using PBE exchange correlation (xc) functional and ultrasoft pseudopotentials from the SSSP library (PBE Precision v1.3.0),^51^ with a cutoff energy for the wave functions (density) of 45 (400) Ry. Dispersion corrections were included in an optimized vdW-DF-like scheme.^52^ A vacuum region of about 12 Å was added in the nonperiodic direction to avoid fictitious interactions with system replicas. The atomic positions within the cell were fully relaxed until the forces were less than 10–4 Ry/bohr. The optimized lattice parameter for MoS_2_ is found to be 3.16 Å, in good agreement with experiments; the T1-MoS_2_ distance amounts to about 3.14–3.22 Å.

## Supplementary Material



## Notes

XRD of T6, additional FFT analysis, and PL from SiO_2_/Si (PDF)
