# Supplementary material for: Highly Ordered T6 Organic Semiconductor Networks on MoS2 Nanosheets for Optoelectronic Applications
Source: ACS Appl Nano Mater. 2026 Feb 4;9(6):2812–20. doi: 10.1021/acsanm.5c05073 (PMC12911063; doi:10.1021/acsanm.5c05073)
Supplement: Supplementary file 1 [file an5c05073_si_001.pdf]

SUPPORTING INFORMATION: Highly Ordered T6 Organic Semiconductor Networks on MoS_2 Nanosheets for Optoelectronics Applications

Nicolò Galizia[1], Pasquale Orgiani[2,3], Claudia Cardoso[4], Deborah Prezzi[4], Antonio Cassinese[1,2] and Riccardo Frisenda[5,*]

[1] Physics Department, Università Napoli Federico II, P.le Tecchio 80, 80125 Naples, Italy

[2] CNR-IOM Istituto Officina dei Materiali, I-34149 Trieste, Italy

[3] AREA Science Park, I-34149 Trieste, Italy

[4] CNR-NANO Istituto Nanoscienze, Centro S3, I-41125 Modena, Italy

[5] Physics Department, Sapienza University of Rome, Piazzale Aldo Moro 5, 00185 Rome, Italy

*E-mail: riccardo.frisenda@uniroma1.it

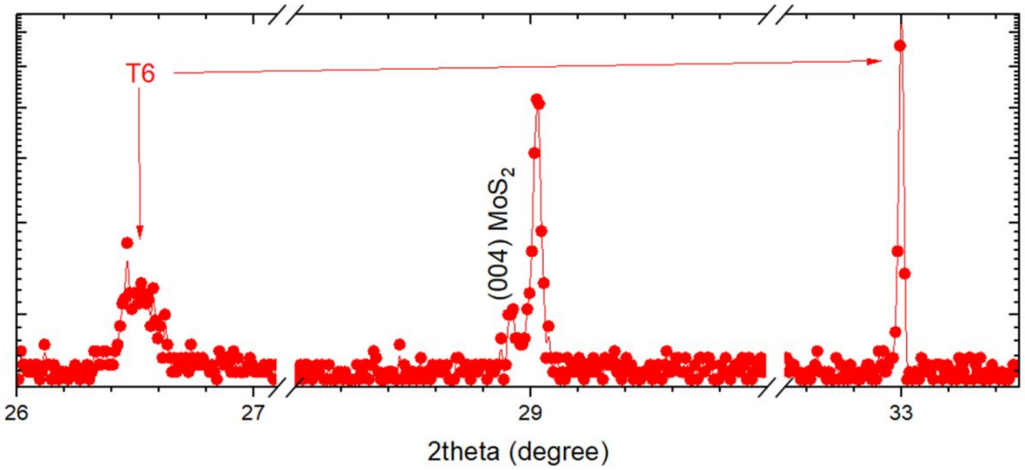

Figure S1: X-ray diffraction from the MoS_2 flake with T6 grown on top. This graph is a zoom in the 26° to 33.5° region from Figure 1b of the main text.

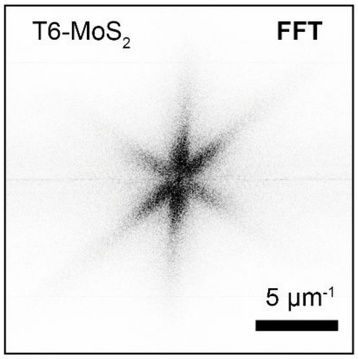

Figure S2: FFT of the AFM topographic image shown in Figure 2a of the main text.

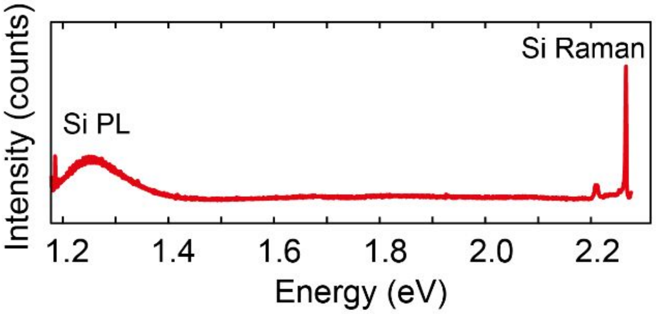

Figure S3: Micro-photoluminescence spectrum at 532 nm excitation of pristine SiO_2/Si substrate.
